# Post-traumatic acute kidney injury: a cross-sectional study of trauma patients

**DOI:** 10.1186/s13049-016-0330-4

**Published:** 2016-11-22

**Authors:** Wei-Hung Lai, Cheng-Shyuan Rau, Shao-Chun Wu, Yi-Chun Chen, Pao-Jen Kuo, Shiun-Yuan Hsu, Ching-Hua Hsieh, Hsiao-Yun Hsieh

**Affiliations:** 1 Department of Trauma Surgery, Kaohsiung Chang Gung Memorial Hospital and Chang Gung University College of Medicine, No.123, Ta-Pei Road, Niao-Song District Kaohsiung City, 833 Taiwan; 2 Department of Neurosurgery, Kaohsiung Chang Gung Memorial Hospital and Chang Gung University College of Medicine, Kaohsiung City, Taiwan; 3 Department of Anesthesiology, Kaohsiung Chang Gung Memorial Hospital and Chang Gung University College of Medicine, Kaohsiung City, Taiwan; 4 Department of Plastic and Reconstructive Surgery, Kaohsiung Chang Gung Memorial Hospital and Chang Gung University College of Medicine, Kaohsiung City, Taiwan

**Keywords:** Trauma, Shock, Acute kidney injury (AKI), Mortality, Length of stay (LOS)

## Abstract

**Background:**

The causes of post-traumatic acute kidney injury (AKI) are multifactorial, and shock associated with major trauma has been proposed to result in inadequate renal perfusion and subsequent AKI in trauma patients. This study aimed to investigate the true incidence and clinical presentation of post-traumatic AKI in hospitalized adult patients and its association with shock at a Level I trauma center.

**Methods:**

Detailed data of 78 trauma patients with AKI and 14,504 patients without AKI between January 1, 2009 and December 31, 2014 were retrieved from the Trauma Registry System. Patients with direct renal trauma were excluded from this study. Two-sided Fisher’s exact or Pearson’s chi-square tests were used to compare categorical data, unpaired Student’s t-test was used to analyze normally distributed continuous data, and Mann–Whitney’s U test was used to compare non-normally distributed data. Propensity score matching with a 1:1 ratio with logistic regression was used to evaluate the effect of shock on AKI.

**Results:**

Patients with AKI presented with significantly older age, higher incidence rates of pre-existing comorbidities, higher odds of associated injures (subdural hematoma, intracerebral hematoma, intra-abdominal injury, and hepatic injury), and higher injury severity than patients without AKI. In addition, patients with AKI had a longer hospital stay (18.3 days vs. 9.8 days, respectively; *P* < 0.001) and intensive care unit (ICU) stay (18.8 days vs. 8.6 days, respectively; *P* < 0. 001), higher proportion of admission into the ICU (57.7% vs. 19.0%, respectively; *P* < 0.001), and a higher odds ratio (OR) of short-term mortality (OR 39.0; 95% confidence interval, 24.59–61.82; *P* < 0.001). However, logistic regression analysis of well-matched pairs after propensity score matching did not show a significant influence of shock on the occurrence of AKI.

**Discussion:**

We believe that early and aggressive resuscitation, to avoid prolonged untreated shock, may help to prevent the occurrence of post-traumatic AKI. However, more evidence is required to support this observation.

**Conclusion:**

Compared to patients without AKI, patients with AKI presented with different injury characteristics and worse outcome. However, an association between shock and post-traumatic AKI could not be identified.

**Electronic supplementary material:**

The online version of this article (doi:10.1186/s13049-016-0330-4) contains supplementary material, which is available to authorized users.

## Background

Renal dysfunction is infrequent, but complicates both the management and the outcome of trauma patients. The incidence of post-traumatic acute kidney injury (AKI) varies widely from 0.1 to 8.4% in published series [1–7] with mortality ranging from 7 to 83% [1, 8–11], and survivors are at increased risk for chronic kidney disease and late death [5, 12–16]. The variation is partly attributed to the controversial definition of terms such as renal insufficiency, renal dysfunction, acute renal failure, and renal failure requiring dialysis, all of which now have been substituted with the term AKI to represent the entire spectrum of acute renal failure [17–19]. However, despite technical progress in the management of AKI over the last 50 years, mortality rates of patients with AKI in critically ill patients still have remained unchanged at around 50% [20].

In early literatures, AKI in trauma patients was reported to be mainly secondary to rhabdomyolysis in crush injuries [11], whereas later decreased renal perfusion was proposed as one of the most common cause of AKI [1, 21]. The shock associated with major trauma may result in inadequate renal perfusion. In addition, simultaneous changes in pro-inflammatory and anti-inflammatory plasma cytokine levels in trauma patients with AKI were found [22]. These circulating factors, such as cytokines and chemokines, activated leukocytes, and adhesion molecules, may lead to distant organ immune cell infiltration and dysfunction [23]. Furthermore, AKI was demonstrated to be neutrophil-mediated during the process of ischemia-reperfusion injury [6]. Therefore, shock in major trauma may cast a detrimental effect on the kidney, which manifests in a variety of outcomes from mild azotemia to severe renal damage that requires renal replacement therapy. In this study, we aimed to investigate the true incidence and clinical presentation of post-traumatic AKI of hospitalized adult patients and its association with shock in a Level I trauma center.

## Methods

### Study design

This retrospective study reviewed all data added to the Trauma Registry System from January 1, 2009 through December 31, 2014 in a 2400-bed facility and Level I regional trauma center that provides care to trauma patients primarily from southern Taiwan. The coding to the Trauma Registry System spanning over these 6 years was performed by same person (Hsu SY). The inclusion criteria were all adult patients (age ≥20 years) hospitalized for treatment of traumatic injury. Patients with incomplete registered data or without complete blood count test as well as blood urea nitrogen (BUN) and creatinine (Cr) test data were excluded from the study. In this study, AKI is defined according to The Kidney Disease: Improving Global Outcomes (KDIGO) Clinical Practice Guidelines [19] as any of the following: increase in serum creatinine by ≥0.3 mg/dL within 48 h; or increase in serum creatinine to ≥1.5 times baseline, which is known or presumed to have occurred within the prior seven days; or urine volume <0.5 mL/kg/h for 6 h. Patients with acute kidney injury (AKI) were identified according to the assigned code 584.9 in ICD-9-CM for AKI, which encompasses all stages. Patients with direct trauma to the kidney or with chronic kidney disease were excluded from the study. We reviewed all 20,106 hospitalized and registered patients added to the Trauma Registry System to compare injury patterns, severity, and mortality of patients with AKI to those patients without AKI. A total of 14,504 adult patients, 78 (0.54%) with AKI and 14,426 (99.46%) without AKI, were enrolled in this study for further analysis. The medical records of these 78 AKI patients had been reviewed to confirm the accuracy of diagnosis and its associated information. Detailed patient information was retrieved from the Trauma Registry System of our institution, including data regarding age, sex, vital signs upon arrival at emergency department (ED), initial Glasgow Coma Scale (GCS) in the emergency department, details of procedures performed at the ED (cardiopulmonary resuscitation, intubation, chest tube insertion, and blood transfusion), Abbreviated Injury Scale (AIS) severity score for each body region, Injury Severity Score (ISS), hospital length of stay (LOS), LOS in ICU, in-hospital mortality, and rates of associated complications. Pre-existing comorbidities and chronic diseases including diabetes mellitus (DM), hypertension (HTN), coronary artery diseases (CAD), congestive heart failure (CHF), and cerebrovascular accident (CVA) were also identified. Blood alcohol concentration (BAC) of 50 mg/dL at the time of arrival to the hospital was defined as a cut-off value and the legal limit for drivers in Taiwan. If required, the doses of intravenous contrast medium Iohexol (OMNIPAQUE) for abdominal contrast-enhanced computed tomography was 60–120 cc, depending on the weight of the patient. The primary analysis was between patients with or without AKI. Odd ratios (ORs) of the associated conditions and injuries of the patients were calculated with 95% confidence intervals (CIs). The data collected were compared using IBM SPSS Statistics for Windows, version 20.0 (IBM Corp., Armonk, NY, USA). Two-sided Fisher’s exact or Pearson’s chi-square tests were used to compare categorical data. Unpaired Student’s t-test was used to analyze normally distributed continuous data, which was reported as mean ± standard deviation. Mann–Whitney’s U test was used to compare non-normally distributed data. To minimize confounding effects due to nonrandomized assignment in the assessment of AKI, propensity scores were calculated by using a logistic regression model and the following covariates: sex; age; comorbidity; GCS; injuries to the head/neck, thorax, abdomen, or extremities based on AIS; and ISS. A 1:1 matched study group was created by the Greedy method with NCSS software (NCSS 10, NCSS Statistical software, Kaysville, Utah). After amending these confounding factors, binary logistic regression was used in the evaluation of interventional factor of shock, which was defined as SBP <90 mmHg, on the occurrence of AKI. *P*-values < 0.05 were considered statistically significant.

## Results

### Characteristics of patients with AKI

The mean age of patients with AKI and patients without AKI were 62.9 ± 21.0 and 52.7 ± 19.2 years, respectively (Table 1). A statistically significant predominance in the percentage of men was found in patients with AKI; of the total 78 patients with AKI, 56 (71.8%) were men and 22 (28.2%) were women. Significantly higher incidence rates of pre-existing comorbidities and chronic diseases including DM (OR, 2.0; 95% CI, 1.22–3.40; *P* = 0.005), HTN (OR, 2.3; 95% CI, 1.47–3.58; *P* < 0.001), CAD (OR, 3.7; 95% CI, 1.83–7.41; *P* = 0.001), and CVA (OR, 2.4; 95% CI, 1.10–5.24; *P* = 0.035) were found among patients with AKI than among patients without AKI. There was no significant difference of positive BAC between patients with and without AKI.

**Table 1:** Demographics and injury characteristics of the adult trauma patients with AKI

Variables	AKI *n* = 78	Non-AKI *n* = 14426	*Odds ratio* *(95%)*	*P*
Gender				
Male	56 (71.8)	8267 (57.3)	1.9 (1.16–3.11)	0.010
Female	22 (28.2)	6159 (42.7)	0.5 (0.32–0.86)	0.010
Age	62.9 ± 21.0	52.7 ± 19.2	—	<0.001
Comorbidity				
DM	20 (25.6)	2087 (14.5)	2.0 (1.22–3.40)	0.005
HTN	36 (46.2)	3928 (27.2)	2.3 (1.47–3.58)	<0.001
CAD	9 (11.5)	494 (3.4)	3.7 (1.83–7.41)	0.001
CVA	7 (9.0)	569 (3.9)	2.4 (1.10–5.24)	0.035
Alcohol >50, n (%)	4 (5.1)	989 (6.9)	0.7 (0.27–2.01)	0.547
GCS	11.5 ± 4.8	14.3 ± 2.3	—	<0.001
≤8	20 (25.6)	718 (5.0)	6.6 (3.94–11.00)	<0.001
9–12	8 (10.3)	557 (3.9)	2.8 (1.36–5.94)	0.011
≥13	50 (64.1)	13151 (91.2)	0.2 (0.11–0.28)	<0.001
AIS, n (%)				
Head/Neck	35 (44.9)	3766 (26.1)	2.3 (1.47–3.61)	<0.001
Face	11 (14.1)	2158 (15.0)	0.9 (0.49–1.77)	0.832
Thorax	17 (21.8)	1610 (11.2)	2.2 (1.29–3.81)	0.003
Abdomen	11 (14.1)	851 (5.9)	2.6 (1.38–4.97)	0.006
Extremity	41 (52.6)	10792 (74.8)	0.4 (0.24–0.58)	<0.001
ISS	19.8 ± 17.3	8.9 ± 7.2	—	<0.001
<16	40 (51.3)	12119 (84.0)	0.2 (0.13–0.31)	<0.001
16–24	15 (19.2)	1599 (11.1)	1.9 (1.09–3.36)	0.023
≥25	23 (29.5)	708 (4.9)	8.1 (4.95–13.26)	<0.001
Mortality, n (%)	35 (44.9)	295 (2.0)	39.0 (24.6–61.8)	<0.001
LOS (days)	18.3 ± 16.8	9.8 ± 10.4	—	<0.001
ICU				
Patients, n (%)	45 (57.7)	2744 (19.0)	5.8 (3.70–9.12)	<0.001
LOS in ICU (days)	18.8 ± 19.7	8.6 ± 11.5	—	0.001

### Injury severity and outcome among patients with AKI

GCS scores were significantly lower in patients with AKI than in patients without AKI (11.5 ± 4.8 vs. 14.3 ± 2.3; *P* < 0.001). There were significantly more patients with AKI that had a GCS ≤8 and GCS of 9–12 compared to those of patients without AKI. Analysis of AIS revealed that patients with AKI had sustained significantly higher rates of head/neck injury, thoracic injury, and abdomen injury than patients without AKI, while patients without AKI had sustained significantly higher rates of extremity injury. A significantly higher ISS was found in patients with AKI than patients without AKI (19.8 ± 17.3 vs. 8.9 ± 7.2, respectively; *P* < 0.001). When stratified by ISS (<16, 16–24 or ≥25), fewer patients with AKI had an ISS of <16 than patients without AKI did. In contrast, more patients with AKI had had an ISS of 16–24 or an ISS ≥25 than patients without AKI. Patients with AKI had a significantly higher mortality than patients without AKI (OR, 39.0; 95% CI, 24.69–61.8; *P* < 0.001). In addition, patients with AKI had significantly longer hospital LOS than patients without AKI (18.3 days vs. 9.8 days, respectively; *P* < 0.001). A higher proportion of patients with AKI than patients without AKI were admitted to the ICU (57.7% vs. 19.0%, respectively; *P* < 0.001), and the LOS in the ICU was greater for patients with AKI compared to patients without AKI (18.8 days vs. 8.6 days, respectively; *P* = 0.001). Of the 2789 trauma patients who were admitted into the ICU during this 6-year span of study, 45 (2.1%) patients had sustained AKI. In this study, 3 of 78 (3.8%) patients with AKI required a renal replacement therapy during the hospitalization.

### Physiological response and procedures performed in the emergency room

As shown in Table 2, patients with AKI exhibited higher ORs for presenting to the emergency room with worse measures of consciousness level (OR, 5.8; 95% CI, 3.62–9.21; *P* < 0.001), systolic blood pressure (OR, 3.5; 95% CI, 1.53–8.19; *P* = 0.010), heart rate (OR 2.4; 95% CI, 1.52–3.85; *P* < 0.001), and respiratory rate (OR, 9.6; 95% CI, 3.42–26.80; *P* = 0.001) in comparison with patients without AKI. Significantly lower hemoglobin (Hb) and hematocrit (Hct) levels, as well as higher BUN (28.9 ± 24.5 vs. 15.5 ± 10.0; *P* < 0.001) and Cr (2.1 ± 2.2 vs. 1.1 ± 1.8; *P* < 0.001) of the blood was found in patients with AKI than patients without AKI. In addition, patients with AKI had higher odds of requiring procedures, including cardiopulmonary resuscitation, intubation, chest tube insertion, and blood transfusion, in the emergency department than patients without AKI. There was no difference of SBP measured just before leaving ER (143.9 ± 41.1 vs. 140.0 ± 27.1; *p* = 0.402) and of the time spent at ER in the patients with AKI and without AKI (4.5 ± 4.7 vs. 4.2 ± 4.7; *p* = 0.566), albeit the exact time of a profound shock at the ER was unknown.

**Table 2:** Significant associated injuries among the trauma patients with AKI

Variables	AKI *n* = 78	Non-AKI *n* = 14426	*Odds ratio* *(95%)*	*P*
Physiology at ER, n (%)				
GCS <13	28 (35.9)	1275 (8.8)	5.8 (3.62–9.21)	<0.001
SBP <90 mmHg	6 (7.7)	332 (2.3)	3.5 (1.53–8.19)	0.010
Heart rate >100 beats/min	28 (35.9)	2714 (18.8)	2.4 (1.52–3.85)	<0.001
Respiratory rate <10 or >29	4 (5.1)	81 (0.6)	9.6 (3.42–26.80)	0.001
Hemoglobin (Hb)	12.1 ± 2.9	13.2 ± 2.1	—	0.001
Hematocrit (Hct)	35.8 ± 7.9	39.1 ± 5.6	—	<0.001
BUN	28.9 ± 24.5	15.5 ± 10.0	—	<0.001
Creatinine (Cr)	2.1 ± 2.2	1.1 ± 1.8	—	<0.001
Procedures at ER, n (%)				
Cardiopulmonary resuscitation	4 (5.1)	21 (0.1)	37.1 (12.42–110.66)	<0.001
Intubation	12 (15.4)	368 (2.6)	6.9 (3.72–12.96)	<0.001
Chest tube insertion	4 (5.1)	184 (1.3)	4.2 (1.51–11.56)	0.019
Blood transfusion	13 (16.7)	528 (3.7)	5.3 (2.88–9.61)	<0.001
SBP before leaving ER (mmHg)	143.9 ± 41.1	140.0 ± 27.1	—	0.402
Time stayed in ER (hour)	4.5 ± 4.7	4.2 ± 4.7	—	0.566

### Associated injuries among patients with AKI

Additional file 1: Table S1 shows the incidence of associated injuries in patients with and without AKI. A significantly higher percentage of patients with AKI had sustained subdural hematoma (OR, 1.8; 95% CI, 1.00–3.32; *P* = 0.045), intracerebral hematoma (OR, 3.8; 95% CI, 1.66–8.91; *P* = 0.007), intra-abdominal injury (OR, 4.3; 95% CI, 1.72–10.75; *P* = 0.008), hepatic injury (OR, 3.3; 95% CI, 1.18–9.00; *P* = 0.040), and rhabdomyolysis (OR, 183.1; 95% CI, 68.65–488.23; *P* < 0.001) (Table 3).

**Table 3:** Physiological response and procedures performed upon arrival at the emergency department

Variables	AKI *n* = 78	Non-AKI *n* = 14426	*Odds ratio* *(95%)*	*P*
Subdural hematoma (SDH)	13 (16.7)	1424 (9.9)	1.8 (1.00–3.32)	0.045
Intracerebral hematoma (ICH)	6 (7.7)	306 (2.1)	3.8 (1.66–8.91)	0.007
Intra-abdominal injury	5 (6.4)	226 (1.6)	4.3 (1.72–10.75)	0.008
Hepatic injury	4 (5.1)	235 (1.6)	3.3 (1.18–9.00)	0.040
Rhabdomyolysis	8 (10.3)	9 (0.1)	183.1 (68.65–488.23)	<0.001

### Association of shock with occurrence of AKI

After propensity score matching with a 1:1 ratio, 71 well-balanced pairs of patients were used for outcome comparison (Table 4). In these propensity score-matched patients, there was no significant difference in sex; age; co-morbidity (DM, HTN, CAD, and CVA); GCS; injury to head/neck, thorax, abdomen or extremities based on AIS; and ISS. Logistic regression analysis did not show that shock, under the definition of SBP <90 mmHg, significantly influenced the occurrence of AKI (OR, 0.8; 95% CI, 0.24–2.82; *P* = 0.754).

**Table 4:** Significant covariates of the trauma patients with and without AKI before and after propensity score matching (1:1 greedy matching)

	Before matching				After matching			
Variables	AKI *n* = 78	Non-AKI *n* = 14426	*Odds ratio* *(95%)*	*P*	AKI *n* = 71	Non-AKI *n* = 71	*Odds ratio* *(95%)*	*P*
Gender								
Male	56 (71.8)	8267 (57.3)	1.9 (1.16–3.11)	0.010	51 (71.8)	51 (71.8)	1.0 (0.48–2.08)	1.000
Female	22 (28.2)	6159 (42.7)	0.5 (0.32–0.86)	0.010	20 (28.2)	20 (28.2)		
Age	62.9 ± 21.0	52.7 ± 19.2	—	<0.001	62.9 ± 21.4	62.3 ± 21.4	—	0.869
Comorbidity								
DM	20 (25.6)	2087 (14.5)	2.0 (1.22–3.40)	0.005	18 (25.4)	18 (25.4)	1.0 (0.47–2.13)	1.000
HTN	36 (46.2)	3928 (27.2)	2.3 (1.47–3.58)	<0.001	31 (43.7)	31 (43.7)	1.0 (0.52–1.94)	1.000
CAD	9 (11.5)	494 (3.4)	3.7 (1.83–7.41)	0.001	7 (9.9)	7 (9.9)	1.0 (0.33–3.02)	1.000
CVA	7 (9.0)	569 (3.9)	2.4 (1.10–5.24)	0.035	5 (7.0)	5 (7.0)	1.0 (0.28–3.62)	1.000
GCS	11.5 ± 4.8	14.3 ± 2.3	—	<0.001	11.9 ± 4.6	12.1 ± 4.6	—	0.784
AIS, n (%)								
Head/Neck	35 (44.9)	3766 (26.1)	2.3 (1.47–3.61)	<0.001	34 (47.9)	34 (47.9)	1.0 (0.52–1.93)	1.000
Thorax	17 (21.8)	1610 (11.2)	2.2 (1.29–3.81)	0.003	14 (19.7)	14 (19.7)	1.0 (0.44–2.29)	1.000
Abdomen	11 (14.1)	851 (5.9)	2.6 (1.38–4.97)	0.006	10 (14.1)	10 (14.1)	1.0 (0.39–2.57)	1.000
Extremity	41 (52.6)	10792 (74.8)	0.4 (0.24–0.58)	<0.001	40 (56.3)	40 (56.3)	1.0 (0.52–1.94)	1.000
ISS	19.8 ± 17.3	8.9 ± 7.2	—	<0.001	18.3 ± 15.2	18.1 ± 15.2	—	0.960
SBP <90 mmHg	6 (7.7)	332 (2.3)	3.5 (1.53–8.19)	0.010	5 (7.0)	6 (8.5)	0.8 (0.24–2.82)	0.754

## Discussion

This study compared the characteristics of injuries and outcome observed in a broad group of trauma patients with AKI to those of patients without AKI hospitalized at a Level I trauma center. Patients with AKI presented with a significantly older age, higher incidence rates of pre-existing comorbidities, different bodily injury patterns, and higher injury severity than patients without AKI. In addition, patients with AKI had longer hospital and ICU stays, higher proportion of admission into the ICU, and higher short-term mortality. However, logistic regression analysis of well-matched pairs after propensity score matching did not show a significant influence of shock on the occurrence of AKI.

In a multi-center cohort study of 1044 trauma patients in the ICU, renal failure developed in 3.5% of patients within 24 h [24]. In a large multinational, multicenter observational study involving over 29,000 critically ill patients, the prevalence of AKI was 5.7% [2]. Post-traumatic renal failure was reported to develop in 323 (1%) of 33,376 trauma patients, with an overall mortality of 38% (*n* = 120) [9]. In this study, the incidence of AKI was 78 (0.54%) of 14,504 hospitalized adult patients, and 45 (2.1%) of 2789 patients in the ICU had sustained AKI. It has been reported that approximately one third of patients with posttraumatic AKI may be a result of inadequate resuscitation [1] and that the prognosis of posttraumatic AKI is adversely influenced by hypotension [25]. However, we did not identify a significant association of shock on the occurrence of AKI in the analysis of well-matched pairs. The causes of post-traumatic renal failure are multifactorial [10, 26, 27]. In addition to hypovolemia, predisposing risk factors such as diabetes and hypertension, pre-existing renal impairment, sepsis, and nephrotoxins, such as aminoglycoside antibiotics and radiological contrast agents, contribute to AKI [9, 26]. In addition, patients sustained renal failure because of prolonged untreated shock and aggressive resuscitation is recommended for such patients [25]. During recent decades, the dramatic increase in intravenous fluid administration to trauma patients during the first 24 h after injury has markedly reduced the incidence of AKI and has improved the outcome [28]. The change in volume therapy helped to nearly eliminate AKI from 8.4 to 3.7% [28]. The reduction of the time under a profound shock may be the key to prevent the occurrence of AKI in a trauma injury. In addition to adequate hydration of major trauma patients with shock, we believe that early identification of patients requiring resuscitation in our hospital [29, 30] and a shorter transport time from the injury scene [31] also might contribute to minimize complications such as AKI. However, the various trauma patient populations and injury severity as well as different indications for hospitalization and admission into the ICU make further comparison impossible. Furthermore, the exclusion of renal trauma patients from this study may reduce the incidence of AKI, albeit subsequent various outcomes depend on the various degrees and types of injury [9].

In this study, patients with AKI had sustained significantly higher rates of head/neck injury, thoracic injury, and abdomen injury, but lower rates of extremity injury than patients without AKI. In addition, a significantly higher percentage of patients with AKI had sustained subdural hematoma, intracerebral hematoma, intra-abdominal injury, and hepatic injury. There is increasing evidence in the relationship between abnormal kidney function and spontaneous intracerebral hemorrhage [32]. The use of antihypertensive agents [33] and principle of volume depletion as fluid restriction [34] had been applied in treating the patients with traumatic brain injury. In addition, it has been reported that an aggressive systolic blood pressure reduction in patients with intracerebral hemorrhage may precipitate acute renal injury [34]. However, the mechanism behind the association of post-traumatic AKI and subdural hematoma or intracerebral hematoma is unknown and yet to be determined. Furthermore, it has been reported that elevated intra-abdominal pressure during trauma is also a significant determinant for the impairment of renal function by direct renal compression and is not related to cardiac output [35]. An association of AKI and hepatic injury was identified in this study. Although the pathophysiological mechanisms underlying AKI in hepatic injury is unknown, it has been reported that AKI is associated with liver ischemia/reperfusion injury, which would evoke substantial systemic inflammatory responses and subsequent distant organ injury, such as to the kidney [36, 37]. Furthermore, independent predictors of mortality in patients with AKI included the severity of cardiovascular, hepatic, and neurologic dysfunction, highlighting the critical interaction between the kidney and remote organ systems [38]. In addition, although rhabdomyolysis is a recognized complication of traumatic injury to be associated with the development of AKI [39, 40], in this study, only 8 of 78 patients with AKI had a rhabdomyolysis. Therefore, rhabdomyolysis may only explain the development of AKI in only a few proportion of patients with AKI.

There are some limitations in this study. This retrospective design with its inherent selection bias results in a major limitation of the current study and makes it impossible to fully account for potential confounders of important risk factors. This includes a history of kidney disease other than CKD, contrast-induced nephropathy, rhabdomyolysis, left ventricular ejection fraction, perioperative and overall blood product transfusions, initial administered fluid amount, the type and duration of use of potential nephrotoxic agents, emergency surgery, type of surgery, and most important, the duration of shock and the associated lactate and base excess level at the ED. Further, the descriptive study design prevents assessment of the effects of any particular treatment intervention, and could only rely on the assumption of uniform assessment and management of patients with or without AKI. The comparison of only the initial Cr level but not a serial of change of Cr or a measured glomerular filtration rate (GFR) level may not reflect well a dynamic renal function. The time lag before Cr starts to rise after tubular/glomerular injury and the dilution effect of volume resuscitation after trauma injury can lead to a selection bias and some cases of AKI might have been missed. Patients declared dead upon hospital arrival or at the accident scene were not included in the Trauma Registry Database and the outcomes that included late mortality was not analyzed, which potentially biases the assessment of mortality.

## Conclusion

Patients with post-traumatic AKI presented with significantly older age, higher incidence rates of pre-existing comorbidities, different bodily injury patterns, and higher injury severity than those patients without AKI. These patients with post-traumatic AKI had longer hospital and ICU stays, higher proportion of patients admitted to the ICU, and higher mortality than those without AKI. However, an association of shock with post-traumatic AKI could not be identified.

## Additional file

Additional file 1: Table S1. Associated injuries among the trauma patients with or without AKI. (DOCX 17 kb)

## Abbreviations

AIS: Abbreviated injury scale
AKI: Acute kidney injury
BAC: Blood alcohol concentration
BUN: Blood urea nitrogen
CAD: Coronary artery diseases
CHF: Congestive heart failure
CIs: Confidence intervals
Cr: Creatinine
CVA: Cerebrovascular accident
DM: Diabetes mellitus
ED: Emergency department
GCS: Glasgow coma scale
GFR: Glomerular filtration rate
Hb: Hemoglobin
Hct: Hematocrit
HTN: Hypertension
ICU: Intensive care unit
IRB: Institutional review board
ISS: Injury severity score
KDIGO: Kidney disease: improving global outcomes
LOS: Hospital length of stay
OR: Odds ratio
