# Supplementary material for: Post-traumatic acute kidney injury: a cross-sectional study of trauma patients
Source: Scand J Trauma Resusc Emerg Med. 2016 Nov 22;24:136. doi: 10.1186/s13049-016-0330-4 (PMC5120453; doi:10.1186/s13049-016-0330-4)
Supplement: Additional file 1: Table S1. — Associated injuries among the trauma patients with or without AKI. (DOCX 17 kb) [file 13049_2016_330_MOESM1_ESM.docx]

**Supplementary Table**

| Variables | AKI  n=78 | Non-AKI  n=14426 | *Odds ratio*  *(95%)* | *P* |
| --- | --- | --- | --- | --- |
| Head trauma, n (%) |  |  |  |  |
| Neurologic deficit | 0(0.0) | 92(0.6) | — | 1.000 |
| Cranial fracture | 6(7.7) | 788(5.5) | 1.4(0.63-3.33) | 0.322 |
| Epidural hematoma (EDH) | 3(3.8) | 549(3.8) | 1.0(0.32-3.22) | 1.000 |
| Subdural hematoma (SDH) | 13(16.7) | 1424(9.9) | 1.8(1.00-3.32) | 0.045 |
| Subarachnoid hemorrhage (SAH) | 12(15.4) | 1349(9.4) | 1.8(0.95-3.27) | 0.068 |
| Intracerebral hematoma (ICH) | 6(7.7) | 306(2.1) | 3.8(1.66-8.91) | 0.007 |
| Cerebral contusion | 7(9.0) | 738(5.1) | 1.8(0.84-3.99) | 0.121 |
| Cervical vertebral fracture | 2(2.6) | 136(0.9) | 2.8(0.67-11.37) | 0.170 |
| Maxillofacial trauma, n (%) |  |  |  |  |
| Orbital fracture | 1(1.3) | 204(1.4) | 0.9(0.13-6.54) | 1.000 |
| Nasal fracture | 1(1.3) | 130(0.9) | 1.4(0.20-10.35) | 0.508 |
| Maxillary fracture | 4(5.1) | 723(5.0) | 1.0(0.37-2.81) | 0.797 |
| Mandibular fracture | 3(3.8) | 270(1.9) | 2.1(0.66-6.69) | 0.182 |
| Thoracic trauma, n (%) |  |  |  |  |
| Rib fracture | 6(7.7) | 1106(7.7) | 1.0(0.44-2.31) | 0.993 |
| Sternal fracture | 1(1.3) | 21(0.1) | 8.9(1.18-67.06) | 0.112 |
| Hemothorax | 4(5.1) | 225(1.6) | 3.4(1.24-9.41) | 0.035 |
| Pneumothorax | 3(3.8) | 202(1.4) | 2.8(0.88-9.01) | 0.098 |
| Hemopneumothorax | 1(1.3) | 194(1.3) | 1.0(0.13-6.89) | 1.000 |
| Lung contusion | 2(2.6) | 157(1.1) | 2.4(0.58-9.82) | 0.211 |
| Thoracic vertebral fracture | 2(2.6) | 138(1.0) | 2.7(0.66-11.21) | 0.174 |
| Abdominal trauma, n (%) |  |  |  |  |
| Intra-abdominal injury | 5(6.4) | 226(1.6) | 4.3(1.72-10.75) | 0.008 |
| Hepatic injury | 4(5.1) | 235(1.6) | 3.3(1.18-9.00) | 0.040 |
| Splenic injury | 1(1.3) | 128(0.9) | 1.5(0.20-10.51) | 0.503 |
| Retroperitoneal injury | 0(0.0) | 28(0.2) | — | 1.000 |
| Renal injury | 1(1.3) | 67(0.5) | 2.8(0.38-20.30) | 0.308 |
| Urinary bladder injury | 0(0.0) | 1(0.0) | — | 1.000 |
| Lumbar vertebral fracture | 4(5.1) | 248(1.7) | 3.1(1.12-8.52) | 0.047 |
| Sacral vertebral fracture | 0(0.0) | 71(0.5) | — | 1.000 |
| Extremity trauma, n (%) |  |  |  |  |
| Scapular fracture | 1(1.3) | 221(1.5) | 0.8(0.12-6.03) | 1.000 |
| Humeral fracture | 6(7.7) | 705(4.9) | 1.6(0.70-3.74) | 0.282 |
| Radial fracture | 5(6.4) | 1544(10.7) | 0.6(0.23-1.42) | 0.221 |
| Ulnar fracture | 4(5.1) | 750(5.2) | 1.0(0.36-2.70) | 1.000 |
| Metacarpal fracture | 2(2.6) | 362(2.5) | 1.0(0.25-4.18) | 0.723 |
| Pelvic fracture | 3(3.8) | 380(2.6) | 1.5(0.46-4.71) | 0.463 |
| Femoral fracture | 20(25.6) | 2555(17.7) | 1.6(0.96-2.67) | 0.068 |
| Patella fracture | 0(0.0) | 392(2.7) | — | 0.277 |
| Tibia fracture | 7(9.0) | 1048(7.3) | 1.3(0.58-2.74) | 0.562 |
| Fibular fracture | 4(5.1) | 593(4.1) | 1.3(0.46-3.46) | 0.564 |
| Calcaneal fracture | 1(1.3) | 692(4.8) | 0.3(0.04-1.86) | 0.186 |
| Metatarsal fracture | 1(1.3) | 378(2.6) | 0.5(0.07-3.48) | 0.725 |
